# Hospital Meetings, &c.

**Published:** 1899-02-11

**Authors:** 


### HOSPITAL MEETINGS, &C.

### HOSPITAL FOR CHILDREN AND WOMEN, WATERLOO ROAD, S.E.

On Thursday, February 2nd, the annual meeting of the Governors of the Hospital for Children and Women, Waterloo Road, S.E., was held at the Mansion House, Mr. Sheriff Alliston presiding. There were present: The Lady Mayoress, Mr. Sheriff Probyn and Mrs. Probyn, Sir Edward Durning Lawrence, Mr. Topham Richardson, &c. The Chairman expressed his regret that he had been obliged to take the chair for the Lord Mayor, who was suffering from an attack of bronchitis. He was recovering, but not permitted to leave his room, but he had sent a practical expression of his sympathy with the hospital in the shape of a cheque for £10 10s. After the formality of signing the minutes had been completed the secretary read the report for the year ending December 31st, 1898. The hospital had lost by death two old and valued friends in Mr. John F. Eastwood, D.L., J.P., the treasurer for thirty years, and Mr. Thomas Gabriel, who joined the committee forty years ago. Mr. John Topham Richardson has been elected treasurer; and Mr. Seymour Eastwood, eldest son of the late treasurer, has been elected to fill the vacant seat on the committee. Some leases of houses adjoining and belonging to the hospital are due to fall in this year, and will thus afford an opportunity to enlarge the building and erect a nurses' home. Amongst the legacies left to the hospital one of £4,000 from the executors of Mrs. C. L. Armitage has been invested in freehold property in Mortlake and Surbiton. The festival dinner at the Hotel Cecil produced nearly £500, whilst the Lambeth Diamond Jubilee Cot Fund reached £236, and has been invested in Indian Three per Cent. stock. The "Annie Louise" bed is maintained by an annual subscription of £31 10s. from the near kin of Annie Louise Walpole; and the four sons of the late Mr. Eastwood have permanently endowed the Frances Mary Eastwood Cot No. 1, which will henceforth bear the name of the John F. and Frances Mary Eastwood Cot No. 1. The grants from the Hospital Saturday and Sunday Funds show a decrease due to the diminished incomes of those funds, and not, the committee are assured, to any shortcomings on the part of the hospital management. No grant was received from the Prince of Wales's Fund, as it is limited to hospitals of 100 beds and over. The suggestions, however, of the committee of inspection as to various improvements have been carried out, and the expense defrayed by a special donation of £100. For three months in 1897 the hospital was closed for repairs, consequently there is this year a marked increase in the number of cases and attendances, as compared with the number during that year. The increase for 1898 is, however, still appreciable when the numbers are compared with 1896. The ordinary income of the hospital is £2,000; the expenditure is about £4,000. Even with the strictest economy, therefore, £2,000 more is needed every year to make both ends meet. The liabilities on January 1st amounted to £1,694. The Chairman, in proposing that the report be accepted, said that the hospital was a Royal one. Her Majesty was the president. It was a City charity, and that though all charities connected with the metropolis were City charities, this one was so in a more special way. He accentuated the fact of the extreme poverty in South London. It was due to the intermittent work of the riverside watermen, and the urgent need of the hospital in its present site. The class of patients appealed to everyone. Sickness was distressing in all, but what could be more pathetic than the sufferings of women and children? The enlargement of the hospital was needed, suitable accommodation for the nurses required, and he appealed to the meeting, and through the meeting to the public, for support. He trusted to the Press to carry the appeal much further than they could do in the ordinary reports. Sir Edwin D. Lawrence, in seconding the resolution, paid a tribute of appreciation to the memory of the late treasurer, and congratulated the governors upon securing the services of Mr. Topham Richardson, who was already devoting much time and thought to the finances of the hospital. He was sure that the public could not do wrong in giving them a little money to spend for the benefit of the patients resorting to this hospital. The usual votes of thanks were carried unanimously, and the collection in the room reached the unprecedented sum in the annals of the hospital of about £620: The Mayor, £10 10s.; Mr. Sheriff Alliston and Probyn, each £5 5s.; Mr. J. W. Simmons, £10 10s.; L. H. Hamilton, £500; Sir E. D. Lawrence, £10 10s.; Lady Lawrence, £5 5s.; the Treasurer, £10 10s.; Miss D. Smith, £10 10s.; the Rev. M. S. and Mrs. Edgell, £20; Sir Frederick Wigan, £10 10s.; Lady Wigan, £5 5s.; Miss Giles, £5 5s.; Miss Gordon Clarke, £5 5s., &c.

### HAMPSTEAD HOSPITAL.

A very enthusiastic gathering in aid of the Hampstead Hospital Building Fund was held on Saturday afternoon last (4th inst.) at the Conduit Lodge, Fitzjohn's Avenue, Hampstead, by invitation of Mr. and Mrs. Wightman, Sir Richard Temple presiding.

Mr. R. A. Owthwaite, the hon. secretary, at the commencement of the proceedings, read the following letter from Sir Henry Burdett, who he was sure they would all have been glad to see had he been able to be present as was expected. Sir Henry wrote:—

"I much regret that I am prevented from attending your meeting, as I am greatly interested in Hampstead Hospital, a fact of which I am sure I need not assure you. I am convinced that your present hospital is not situated in a convenient spot to serve the needs of so wide-spreading an area as Hampstead. For this reason I am in favour of the proposal for a new building. Your present hospital contains 32 beds, and although not all of them have been continuously occupied, Hampstead is rapidly growing larger, and in my view you will do well to provide for the future to the extent of slightly increased accommodation now—by far the most economical plan when building operations are undertaken initially. I think that with regard to your new hospital 50 beds should be sufficient; and if you expend £200 to £250 per bed upon your buildings and equipment you should, for the sum of from £10,000 to £12,500, have a hospital worthy of so important a part of London as Hampstead. This is so small a sum to ask of so rich a population as Hampstead that I have no doubt Mr. Malcolm, your treasurer, will not have to wait long before he has the total amount in his possession."

Letters were also read from Dr. Edmund Gwynn, Medical Officer of Health for Hampstead, and others, expressing approval of the scheme.

The Chairman, in explaining the purpose of the meeting, briefly referred to the fact that the population of that great suburb was separated from the nearest of the existing hospitals by prohibitory distances. They had, of course, a small local hospital, wonderfully well managed, but it was situated in a corner of the parish, and the structure was quite unsuited to its purpose, being simply several private houses knocked into one. The question of the new site must be carefully considered. Their friend Mr. Parker had been investigating a number of sites, and the choice of one from among these would have to be made by an informal committee. They would, he supposed, have a fund for the site and a separate one for the building. It would certainly be desirable to secure the land as quickly as possible, for if they waited till the whole of the money required had been raised they might find that some of the best sites had passed out of the market, whereas, the land once secured, they could if necessary wait a year or so while the money for the building was being got together. Having thus introduced the subject, the Chairman called upon Mr. Edmund Owen, one of the consulting surgeons of the hospital, to move the first resolution.

Mr. Owen made an eloquent speech, the clearness and forcefulness of which, as well as its effective touches of humour, were obviously appreciated by the audience. He began by expressing regret at the absence of Sir Henry Burdett, but remarked that the fact that he had wished to come, and had written the letter which had been read, were a sort of testimonial to the soundness of their cause. Continuing, the speaker went on to draw a comparison between the view to be obtained looking over Hampstead with that he had seen on the plain of Umbria, where, in the mediæval city of Perugia, there were the same red-tiled roofs among the trees, though those in Perugia had been mellowed and toned down by four centuries of weather. Both places were noted for their artists, and the landscapes were not at all unlike—the water of the Welsh Harp in the distance standing for the Tiber in flood. But there the comparison broke down, for while Perugia, with a population of 17,500, had a first-rate hospital, equipped with all modern improvements, Hampstead, with a population of 70,000, had none, for he could not admit that the three or four ragged houses, smelling of carbolic acid, which they were pleased to dignify by the name constituted a hospital in any proper sense of the word. First, as to its situation. Not long since hurrying along one of the passages there his head came into violent contact with a beam, and though, since one of the hospital sisters was with him, he did not say all he might otherwise have done, he could not help thinking if he were to be laid up with concussion of the brain and irritation of the nervous system, that the noise of the luggage trains immediately below, the brakes, the whistles, and so forth, would very seriously retard the recovery he might be making. What they were pleased to call the hospital on Parliament Hill had clearly been intended for railway men, who would most certainly be at home there, but it was not suited to any other class of patients. Then the accommodation for the staff was most inadequate. He was afraid they had a sort of Box and Cox arrangement by which when the day nurse left her room the night nurse came in, and that was an arrangement which was not even fair to the bed. Another point, medicine and surgery were not yet among the exact sciences; they could not always be sure of curing their cases; suppose one of their patients, suffering, say, from chronic bronchitis, and Anno Domini, died in the night—this was somewhat gruesome, but he wanted to put the matter plainly—what were they to do? Put the body in the mortuary? There was none! They must convey the corpse to the bathroom, or let it lie in the ward, both equally undesirable courses from the point of view of the well-being of the remaining cases. He had said enough, he thought, to show that the present state of things was bad for the staff, for the nurses, and for the patients. Then as to the remedy. It was objected that if they started building there would be a great waste of money, and no one would know where it would stop; but he was assured that the Ladies' Association had met, and solemnly bound themselves that the building they were going to erect should be for 50 beds only; that there should be no extravagance and no waste; and that the new institution should be carried on on the same lines which so far had been so successful. Mr. Owen went on to say that so far as his independent and personal opinion went, and he was careful to emphasise the fact that in this respect he spoke without having consulted the council, and quite possibly in opposition to its opinions, but personally he held strongly that there should not be an outpatient department, which in his view would simply be a big scheme for pauperising those who came to it, and would prove itself an Old Man of the Sea on their backs. As to where the hospital was to be that could be settled later on, but certainly not over a railway cutting. And wherever it was let it be worthy of the social position, and of the geographical position of the borough. It should, he thought, stand four-square to all the winds that blow, with the entrance and the kitchens on the north, and open to the south and west, with suitable creepers growing over it, and lending beauty to every aspect. And over the door he would put the motto *Non sibi sed toti.* He concluded by moving :—

"That the resources of the present hospital, situate on Parliament Hill, being quite inadequate to meet the increasing wants of the growing Borough of Hampstead, the council be requested to take steps as soon as possible to obtain a site in a more convenient position of the parish, on which to build a new General Hospital."

Sir SAMUEL WILKS seconded the resolution. As consulting physician, he urged upon all there the necessity of making the new institution worthy of the very distinguished and very affluent suburb of London in which they lived. Since the central hospitals were built London had grown enormously, that there was no doubt whatever as to the necessity of building fresh ones, and these should certainly be among those who needed them. They ought not to have to carry far cases either of accident or of disease, and he hoped that, as a result of that meeting, a hospital would be built which would be a light to all other suburbs.

Mr. CHAS. WIGHTMAN warmly supported the resolution, but was glad that the delightful speeches they had heard spared him the necessity of speaking at any length, feeling sure they would appreciate the awkwardness he felt at doing so in his own home.

The motion, on being put to the meeting, was carried by acclamation and with evident enthusiasm.

Mr. S. FIGGIS moved the second resolution: "That this meeting pledges itself to use its utmost endeavour to carry out the terms of the previous resolution by raising the necessary funds." He pointed out that this was a magnificent opportunity for one of their great landowners to make them a present of a site, thereby not only doing himself a great honour, but also conferring a real and lasting blessing on the borough. They were all agreed as to the necessity for removal, and that a place like Hampstead should have a hospital of its own. But this could not be done without money, and he was inclined to think that £20,000 would be nearer the figure than the £10,000 or £12,000 Sir Henry Burdett had mentioned. However, he felt sure there were plenty of people ready to put their hands in their pockets. There should be no difficulty in finding one hundred men who would subscribe £100 a-piece, and he was quite ready to make one of them. He would just like to say, however, that in spite of what Mr. Owen had said he thought the question of an out-patient department would bear discussion.

Mr. BOND, M.P., L.C.C., spoke in the same sense, and was followed by

Mr. J. S. FLETCHER, J.P., L.C.C., who said he thought he might mention what Sir Henry Burdett's modesty had prevented him from stating in the letter he had sent, namely, that the reason he was unable to be there was that he had been commanded by the Prince of Wales to go to Sandringham, no doubt to confer with him on the same sort of business. As to the need for the new hospital he need only mention that though they had a population of 75,000 to 80,000, and an area capable of containing 200,000, they were still obliged to treat their sick poor in the sick wards of the workhouse. Not only was this undesirable in many ways, but the limit of accommodation had been reached, and could not be enlarged except at a quite prohibitive cost.

Mr. MALCOLM, the hon. treasurer, also spoke. He reminded his hearers that there was in all England no town of anything like their population without a properly equipped hospital. He thought the absence of support hitherto had been largely from want of knowledge of the circumstances; but the Ladies' Association was overcoming that.

This resolution having been carried unanimously, a vote of thanks was proposed to Mr. and Mrs. Wightman for the use of the rooms and for their hospitality—a proposal which met with a very hearty reception. In acknowledging it, Mr. Wightman said the best vote of thanks they could give him and his wife would be to put their names on the list of subscribers. Headed by Mr. Malcolm with £1,000, this already amounted to over £2,000, and before the gathering separated about £2,500 had been promised.

Several other speakers emphasised the various points of the appeal, and a vote of thanks to Sir Richard Temple for presiding brought a most successful meeting to a close.

